# Construction and characterization of a novel miniaturized filamentous phagemid for targeted mammalian gene transfer

**DOI:** 10.1186/s12934-023-02135-w

**Published:** 2023-07-10

**Authors:** Shirley Wong, Salma Jimenez, Roderick A. Slavcev

**Affiliations:** School of Pharmacy, University of Waterloo, Waterloo, Canada

**Keywords:** Filamentous bacteriophage M13, Phagemid, Gene transfer, Non-viral gene delivery, DNA minivector

## Abstract

**Background:**

As simplistic proteinaceous carriers of genetic material, phages offer great potential as targeted vectors for mammalian transgene delivery. The filamentous phage M13 is a single-stranded DNA phage with attractive characteristics for gene delivery, including a theoretically unlimited DNA carrying capacity, amenability to tropism modification via phage display, and a well-characterized genome that is easy to genetically modify. The bacterial backbone in gene transfer plasmids consists of elements only necessary for amplification in prokaryotes, and, as such, are superfluous in the mammalian cell. These problematic elements include antibiotic resistance genes, which can disseminate antibiotic resistance, and CpG motifs, which are inflammatory in animals and can lead to transgene silencing.

**Results:**

Here, we examined how M13-based phagemids could be improved for transgene delivery by removing the bacterial backbone. A transgene cassette was flanked by isolated initiation and termination elements from the phage origin of replication. Phage proteins provided in trans by a helper would replicate only the cassette, without any bacterial backbone. The rescue efficiency of “miniphagemids” from these split origins was equal to, if not greater than, isogenic “full phagemids” arising from intact origins. The type of cassette encoded by the miniphagemid as well as the choice of host strain constrained the efficiency of phagemid rescue.

**Conclusions:**

The use of two separated domains of the f1 *ori* improves upon a single wildtype origin while still resulting in high titres of miniphagemid gene transfer vectors. Highly pure lysates of miniaturized phagemids could be rapidly obtained in a straightforward procedure without additional downstream processing.

**Supplementary Information:**

The online version contains supplementary material available at 10.1186/s12934-023-02135-w.

## Background

Bacteriophage (phage)-mediated gene delivery holds much potential. Phage particles are simplistic protein particles that protect DNA cargo easily functionalized by phage display of cell-specific ligands to target uptake into specific tissues. Filamentous phages such as M13 have shown enormous capacity for the incorporation of genetic cargo and peptide display of functional moieties [1]. Importantly, they do not possess intrinsic tropism for mammalian cells, so they are an attractive option for safe gene transfer into mammalian cells. M13-mediated gene transfer has previously been demonstrated on multiple occasions [2–6].

M13 is one of three Ff phages (f1, fd, and M13) that specifically infect F^+^ *Escherichia coli* [7]. Progeny virions extrude from infected host cells over time without cell lysis. The phage’s simplistic genome encodes all eleven proteins necessary for these activities, which are controlled by signalling structures within a short non-coding intergenic region (IR) [8]. Upon infection, the M13 genome is converted to a double-stranded (ds) DNA episomal replicative factor (RF), whereupon phage gene expression occurs. Both replication of filamentous phage DNA and assembly of phage virion particles are directed by specific hairpin loops within the functional origin (f1 *ori*). Replication is initiated by the phage replication protein, pII, cleaving specifically within the *ori*, facilitating host proteins to replicate of the DNA molecule [9, 10]. Replication continues unidirectionally along the genome through a rolling circle mechanism before terminating at the pII cleavage site again [11]. Single-stranded (ss) episomes are later sequestered for assembly into progeny phage through recognition of the packaging signal (PS), another signal within the *ori*, by the phage assembly complex. Assembled phage progeny are extruded out of the cell over the course of the bacterium’s lifetime.

The presence of the f1 *ori* on a vector (“phagemid”) is sufficient to direct its replication by M13 machinery independent of a plasmid origin [12, 13]. Thus, a mammalian transgene vector can be easily propagated and encapsulated into M13 virion particles. As a phagemid molecule itself typically does not encode any phage proteins, a helper phage is necessary to provide proteins in trans [14]. Numerous helper phages have been constructed for different purposes [15–20]. For example, the common helper phage M13KO7 has a defective phage origin of replication due to an interruption of the f1 *ori* by a kanamycin resistance marker and a p15a *ori* [14]. When present, wildtype f1 *ori* are replicated preferentially over the helper, although M13KO7 itself is not completely deficient in self-replication thanks to its plasmid origin of replication.

Multiple intracellular barriers still need to be overcome for mammalian gene transfer by phages or phagemids. It is now well-known that DNA of bacterial origin can detrimentally reduce transgene expression both due to cytosine-guanine dinucleotide (CpG)-mediated inflammatory silencing and excess DNA bulk impacting nuclear import [21, 22]. Therefore, the presence of CpG motifs on a phagemid vector likely contributes to the lack of transgene expression efficiency and gene silencing. Indeed, the primary immune response against M13 appears to be mediated through the same pathways activated by CpG-rich DNA [23–25].

**Fig. 1:**
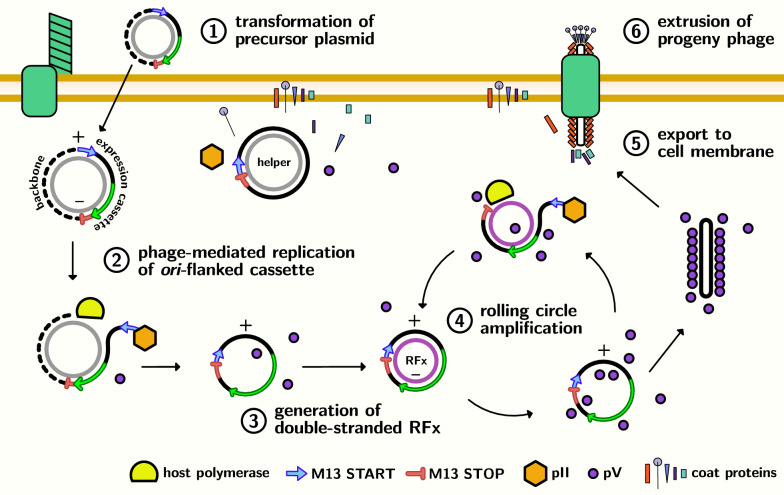
Production and purification of miniphagemid particles. 1) The host cell is transformed by the precursor plasmid encoding a region of interest between the separated M13 replication signals. After infection with a helper phage, phage protein expression can occur. 2) Phage proteins direct the excision of the *ori*-flanked expression cassette, 3) generating a recombinant replicative factor (RFx) that reconstitutes the f1 *ori* and loses the plasmid backbone. 4) The new RFx is amplified through rolling circle amplification. 5) Phage ssDNA binding proteins pV sequester single-stranded minivector, preventing further amplification, and shift the replication cycle towards assembly. 6) Assembly proteins extrude progeny phage particles encapsulating the minivector

Here, we describe the production of miniaturized phagemids (“miniphagemids”), which lack the superfluous and potentially immunostimulatory prokaryotic backbone, for improved gene transfer in mammalian cells. First, we constructed a precursor vector encoding a mammalian transgene cassette flanked by separated signalling domains from the f1 *ori*. As the transgene is encoded between the separated domains, phage-mediated replication is hypothesized to excise and re-circularize the transgene cassette, thereby separating it from the plasmid backbone (Fig. 1). We evaluated if minivectors containing only the transgene cassette could be assembled from such precursors using a commercial helper phage M13KO7 (Table 2). The rescue efficiency of miniphagemids was further characterized using two helper phages (M13KO7 or M13SW8) and six host strains in order to identify optimal host and helper conditions for production.

## Materials and methods

### Strains and vectors

*Escherichia* *coli* K-12 JM109 was used in the generation of all phage and plasmid constructs, where specific strains are listed in Additional file 1: Table S1. Plasmids and phages used or constructed in this study are listed in Tables 1 and 2, respectively. Bacterial strains were cultured in Luria–Bertani (LB) liquid medium. Media were supplemented with the relevant antibiotic as required. Phage lysates were purified and stored in Tris-NaCl (TN) buffer. Where indicated, phage lysates were concentrated with polyethylene glycol (PEG).

**Table 1:** Plasmids used in this study

Plasmid	Genotype	Size (bp)	Source
Sources of genetic cassettes			
pGL2-SS-CMV-GFP-SS	pGL2-Promoter, *cmv-gfp* replaces SV40-luc, AmpR, source of *cmv*-*gfp*	5257	Gift, Mediphage Bioceuticals
pGL3-CMV	pGL3-Basic, *cmv* inserted in BglII-HindIII, AmpR, source of *cmv-luc*	5678	Gift, Dr. N. Oviedo
pBR322	AmpR, TetR, source of *tetA*	4361	[31]
pBluescript and derivatives			
pBluescript II KS +	wildtype f1 *ori*, pUC ori, AmpR	2958	[30]
pSW9	pBluescript II KS + , *cmv-gfp* from pGL2-SS-CMV-GFP-SS inserted in BamHI-EcoRI, AmpR	5019	This study
pSW10	pBluescript II KS + , *cmv-luc* from pGL3-CMV inserted in KpnI, AmpR	5593	This study
pSW9-tet	pSW9, *tetA* from pBR322 inserted into SmaI	6903	This study
pM13ori2 and derivatives			
pM13ori2	split f1 *ori* inserted in pUC57 (Genbank Accession No. Y14837.1), AmpR	2461	This study
pM13ori2.gfp1	pM13ori2, 1 kb stuffer from pGL2-SS-CMV-GFP-SS inserted into BamHI	3803	This study
pM13ori2.cmvgfp	pM13ori2, *cmv-gfp* from pGL2-SS-CMV-GFP-SS inserted in EcoRI-PacI	4480	This study
pM13ori2.cmvluc	pM13ori2, *cmv-luc* from pGL3-CMV inserted in EcoRI-KpnI	5054	This study
pM13ori2.cmvgfp-tet	pM13ori2.cmvgfp, *tetA* from pBR322 inserted into KpnI	6359	This study

**Table 2:** Phages used in this study

Phage	Genotype	Source
Helper phages		
M13KO7	Tn903 (KanR), p15a *ori*	New England BioLabs
M13SW8	M13KO7, PS removed, KanR	This study
Phagemid-carrying phages		
full-(gfp)	From precursor pSW9, AmpR	This study
full-(luc)	From precursor pSW10, AmpR	This study
full-(gfp-tet)	From precursor pSW9-tet, AmpR, TetR	This study
Miniphagemid-carrying phages		
mini-(gfp)	From precursor pM13ori2.cmvgfp, AmpR	This study
mini-(luc)	From precursor pM13ori2.cmvluc, AmpR	This study
mini-(gfp-tet)	From precursor pM13ori2.cmvgfp-tet, AmpR, TetR	This study

### Design and construction of a precursor vector for miniphagemid production

The precursor vector backbone pM13ori2 was designed based on the regions identified by Short et al. [32]. A polylinker was designed in SnapGene 5.3 (https://www.snapgene.com/) and flanked by the separated regions of replication. The cassette was synthesized and subcloned into pUC57 to construct the backbone vector pM13ori2 (Genscript Inc, Piscataway, USA). To construct precursor vectors for miniphagemid production, reporter gene cassettes [encoding either green fluorescent protein (GFP) or luciferase] were subcloned into the polylinker of pM13ori2. To construct precursor vectors for “full” phagemid production, reporter gene cassettes were subcloned into the same backbone carrying both a plasmid and wildtype f1 *ori*: pBluescript II KS +. The cassette *cmv-gfp* from pGL2-SS-CMV-GFP-SS was subcloned directly into the EcoRI-PacI sites of pM13ori2 to generate pM13ori2.cmvgfp, and into the EcoRI-BamHI sites of pBluescript II KS + to generate pSW9. The cassette *cmv-luc* was amplified by PCR to introduce EcoRI-KpnI target sites for subcloning into pM13ori2 to generate pM13ori2.cmvluc and into pBluescript to generate pSW10. To examine the impact of length on miniphagemid production, a fragment from pBR322 was subcloned into the KpnI target site of pM13ori2.cmv-gfp to generate pM13ori2.cmv-gfp-tet and into the SmaI site of pSW9 to generate pSW9-tet. Plasmids are summarized in Table 2 and the sequence of pM13ori2 is provided as Additional file 2: S2.

### Phage amplification and lysate purification

A slightly turbid culture (0.01 < A600 < 0.4) of the host carrying the target phagemid or miniphagemid precursor was infected with helper phage M13KO7 following the manufacturer’s instructions (NEB, Ipswich, USA). The next day, the culture was centrifuged (8000 ×*g*, 10 min) to separate the bacterial pellet containing RF from the supernatant containing the phage lysate. The phage lysate was further purified through a 0.45 μm filter to remove residual bacterial debris. Filtered lysate was concentrated through precipitation with PEG, following the manufacturer’s instructions for M13KO7-rescued phage lysates. They were also treated with DNase I (Promega, Madison, USA) to remove any extraneous phage or bacterial DNA in the sample [33, 34]. The concentrated lysate was stored at 4 °C.

### Purification of the double-stranded RF and single-stranded miniphagemid DNA

Double-stranded (ds) RF DNA were extracted from the pellet with the Monarch Plasmid Miniprep Kit (New England Biolabs). Single-stranded (ss) phagemid DNA were extracted from the phage lysate through phenol–chloroform extraction and ethanol precipitation [35]. Extracted DNA was analyzed on a NanoDrop 2000 to determine concentration and purity. Extracted RFs were linearized with BamHI and visualized via agarose gel electrophoresis (AGE) using Tris–Acetate-EDTA (TAE) buffer, while phagemid ssDNA was visualized without digestion.

### Quantification of phage viability through infectivity assays

Plaque and colony assays were conducted to quantify infective phage particles [36]. For the plaque assay, phage lysates were serially diluted in TN buffer, then mixed with 200 μL aliquots of early log-phase *E. coli* ER2738 (Additional file 1: Table S1). Each aliquot was added to 3 mL top agar supplemented with 5 mM MgSO_4_ and poured on a pre-warmed LB agar plate. Plates were incubated overnight at 37 °C and analyzed the next day. The titre was expressed as plaque forming units (PFU) per millilitre. For the colony assay, phage lysates were prepared from cells doubly transformed by a helper phage [M13KO7 or M13SW8 (Table 2); KanR] and pBluescript II KS + (AmpR). Phage lysates were mixed with a susceptible host as per the procedure for a plaque assay, then the entire aliquot was spread on an LB agar plate supplemented with ampicillin, kanamycin, or both. The titre was expressed as colony forming units (CFU) per millilitre.

### Quantitative PCR of phage lysates

Helper phage and recombinant phagemid genomes within each lysate were quantified through SYBR Green quantitative PCR (qPCR). Calibration curves to quantify the phage were constructed using external standards: M13KE RF (7222 bp; NEB) for helper phage, pGL2-SS-CMV-GFP-SS (5257 bp; Mediphage Bioceuticals, Toronto, Canada) for *gfp*-encoding target phage, and pGL3-CMV (5678 bp) for *luc*-encoding target phage. Primers are summarized in Additional file 1: Table S2. To generate each calibration curve, tenfold serial dilutions of each external control were prepared as templates for the PCR reaction. To prepare the phage particles for PCR, lysates were denatured by heat for 100 °C for 15 min to isolate phage DNA [34]. Ten-fold serial dilutions of each cleared lysate were then prepared as templates for the PCR reaction.

Each 10 μL PCR reaction was prepared using 5 μL of PowerUp SYBR Green Mix (Thermo Fisher Scientific, Waltham, USA), 1 μL each of 500 nM primer (forward and reverse), 2 μL of template, and 1 μL of dH_2_O. PCR cycling conditions were as follows: 50 °C for 2 min, 95 °C for 2 min, followed by 40 cycles at 95 °C for 15 s and 60 °C for 1 min. Next, the melt curve was set for 1 cycle at 95 °C for 15 s, 60 °C for 1 min, and 95 °C for 15 s. PCR reactions were run in triplicate on the StepOne Plus Real-Time PCR system (Applied Biosystems, Waltham, USA). The quantification cycle or threshold cycle number (Cq) for each reaction was used in subsequent analysis.

The following equation was used to convert from mass of dsDNA standard to the number of genome copies [34]:

$$gc=\left(\frac{mass}{size} \times 607.4+157.9\right)\times \left(6.02 \times {10}^{23}\right),$$

where *gc* is the concentration of phage genome copies (genome copies (gc)/μL), *mass* is the mass of the dsDNA standard (g/μL), and *size* is its length (bp). To estimate phage concentrations from their respective calibration curve, the Cq values from each exogenous control were first plotted against the log of the known concentration for each reaction in the dilution series. Linear regression produced an equation of the form:

$$Cq=mx+b,$$

where Cq is the observed threshold cycle number, *m* is the slope, *x* is the base-10 log of the concentration (gc/μL) and *b* is the x-intercept. Subsequently, virion concentration (*V*) was estimated by:

$$V={10}^{(Cq-b)/m} \times 2,$$

where multiplication by 2 adjusts for the estimation of ssDNA products (gc/μL) from dsDNA standards. Only Cq measurements within the bounds of the calibration curve were used to estimate phage concentration.

### Statistical analysis

All statistical analyses were performed using Python (with the packages NumPy 1.22.0 [37], Pandas 1.4.1 [38], SciPy 1.8.0 [39], scikit-bio 0.5.6 [40], and statsmodels 0.13.2 [41]). Values are reported as means of *n* independent experiments with uncertainty reported as the standard deviation (SD), as indicated. Statistical hypothesis tests were evaluated using one-way ANOVA, followed by the Tukey range test for multiple comparisons. Values of *p* < 0.05 were considered statistically significant. The phagemid fraction was determined as the concentration of target phagemid divided by the total virion concentration, expressed as percentages. As compositional data [42], they have a fixed constant sum constraint (100%). In order not to violate this constraint, the data were transformed using an isometric log ratio transformation before performing statistical analyses and transformed back to percentages for reporting.

## Results and discussion

### Production of single-stranded DNA minivector phagemids

**Fig. 2:**
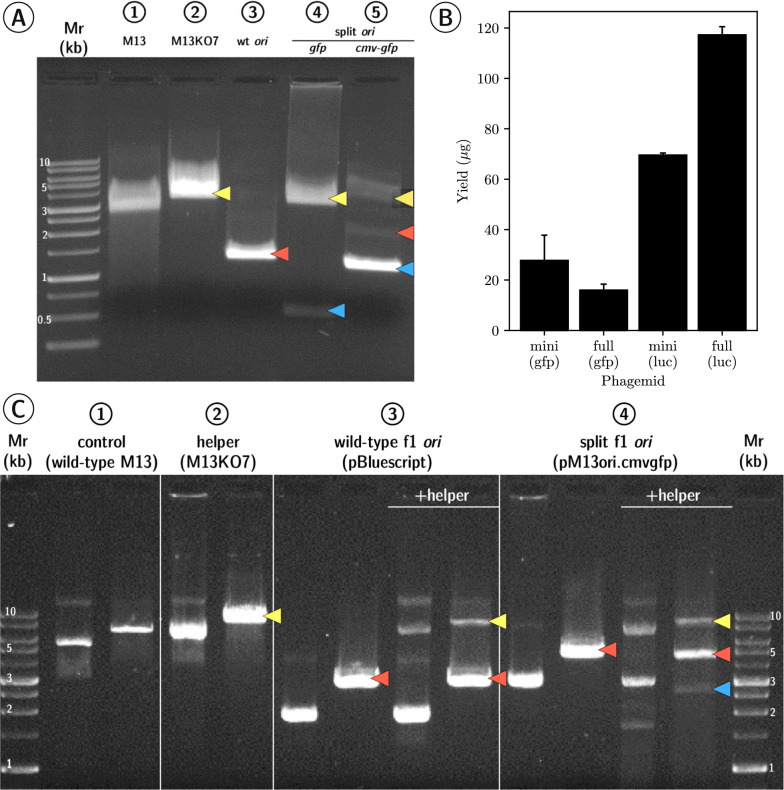
Helper phage can rescue miniaturized phagemids from a split f1 origin. **A** Purified ssDNA runs at approximately half the expected size of the respective dsDNA when visualized via AGE. From left to right, lysates were prepared from: 1) wildtype M13 (6.4 knt), 2) M13KO7 alone (8.7 knt), 3) pBluescript II KS + (3 knt), 4) pM13ori2.gfp1 (plasmid: 3.8 knt, miniphagemid: 1.0 knt), 5) pM13ori2.cmvgfp (plasmid: 4.5 knt, miniphagemid: 2.2 knt). **B** Yield of purified ssDNA was compared across different lysates. Error bars represent SD, *n* = 3. **C** Double-stranded RFs were linearized with BamHI and visualized on AGE. From left to right (uncut, followed by BamHI-cut): 1) wildtype M13 only (6.4 kb), 2) helper M13KO7 alone (8.7 kb), 3) pBluescript II KS + (3 kb) alone or with helper, 4) pM13ori.cmvgfp (4.5 kb) alone or with helper. Arrows indicate bands of interest: yellow (helper genome), red (plasmid or full phagemid), blue (recombinant miniaturized phagemid). Mr: 1 kb ladder (FroggaBio). AGE: 0.8% agarose in TAE, stained with ethidium bromide

We first investigated if filamentous phage M13 was able to produce a new recombinant dsDNA minicircle replicative form (RFx) from a split origin precursor plasmid and assemble this recombinant species into ssDNA minivector progeny virions (miniphagemids) (Fig. 1). In phage lysates, two expected ssDNA species were recovered from helper phage rescue of miniphagemid precursor plasmids: one corresponding to the helper phage itself and the other, the expected miniphagemid (Fig. 2A). Intracellular RF dsDNA were also extracted from helper-infected cells for corroboration (Fig. 2C). In cells transformed by a control phagemid (pBluescript II KS +) and infected by helper phage, two dsDNA species were recovered, as expected: the helper phage genome and the phagemid. In cells only transformed by a miniphagemid precursor without helper phage infection, only the plasmid was recovered, as expected. Upon infection helper phage, an additional DNA species was observed, which corresponded to the predicted size of the hypothesized RFx molecule (2.6 kb). The f1 replication signals can still mediate DNA replication even when f1 signals are distal to each other. This is consistent with observations reported in literature [32, 43]. Thus, miniphagemids could indeed be rescued from a split origin precursor plasmid.

Two mammalian transgene cassettes expressing different reporter genes were subcloned into the polylinker of pM13ori2: *cmv-gfp* (2.1 kb) to generate the miniphagemid mini-(gfp), and *cmv-luc* (2.6 kb) to generate mini-(luc). They were also cloned into the phagemid vector, pBluescript II KS +, which was used as our wildtype f1 *ori* control, to generate “full” phagemid particles full-(gfp) and full-(luc). Miniphagemid rescue from precursor vectors was as robust as the rescue of typical “full” phagemids with an intact wildtype f1 *ori* and an entire bacterial backbone. If we assume only target phagemid was present in the final phage lysate, the overall ssDNA yield was comparable between mini and full phagemid lysates for both cassettes (Fig. 2B). No significant difference was observed in the ssDNA yield arising from a split f1 *ori* compared to an “intact” wildtype *ori*.

However, while the helper phage M13KO7 preferentially packages phagemids over its own interrupted origin, some degree of contaminating helper phage was expected in M13KO7-prepared phage lysates. Presence of the helper phage genome in extracted mini-(gfp) ssDNA (Fig. 2A) suggested to us that the helper phage M13KO7 may rescue the wildtype f1 *ori* preferentially over our split f1 *ori*. We sought to verify this by quantifying phage species present per lysate using qPCR. DNA quantification approaches such as qPCR have been established as highly accurate methods for the quantification of viral genomes, the equivalent to counting the number of virion particles since every virion encapsulates one phage genome [44–46].

### Phagemid rescue efficiency may be dependent on phagemid sequence

**Fig. 3:**
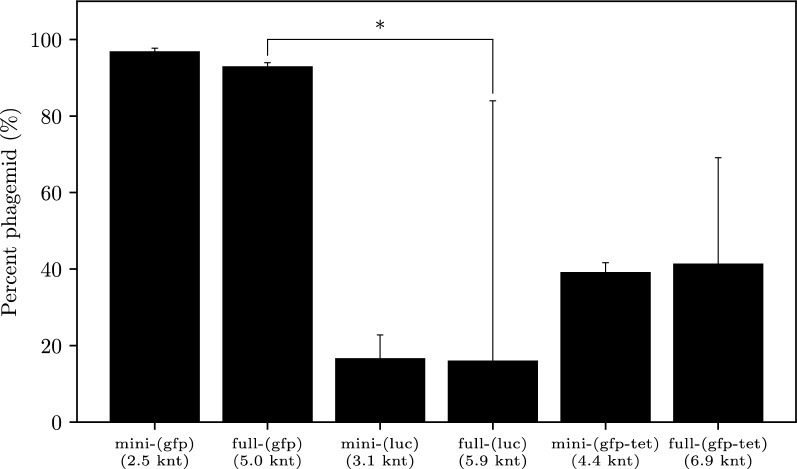
Composition of phage lysates differs between phagemids encoding three different transgene cassettes. The proportion of the target phagemid is shown as a percentage of the total phage population. Error bars represent SD, *n* = 3. The ∗ above the bars indicates a difference at significance level *p* < 0.05

Phagemid rescue efficiency has previously been linked to phagemid length [13]. Levinson et al. (1984) observed poor conversion of large phagemids (5–10 kb) from intracellular RF to extracellular phage-encapsulated DNA. To investigate this, a longer transgene cassette was also constructed through the addition of the TetR fragment from pBR322 to our *cmv-gfp* cassette: the corresponding miniphagemid, mini-(*gfp-tet*), was expected to be 4.4 knt long (Table 2). If phagemid length size did affect production efficiency, mini-(*gfp-tet*) and, especially, full-(*gfp-tet*) (6.9 knt) would be extruded less efficiently than mini-(*luc*) (3.1 knt) and full-(luc) (5.9 knt). Rescue efficiency of the commercial helper phage M13KO7 was then evaluated in the production of miniaturized phagemids (miniphagemids) encoding one of the three transgene cassettes (*cmv-gfp*, *cmv-luc*, and *cmv-gfp-tet*) in comparison with their full phagemid counterparts (Fig. 3). Both mini-(*gfp*) (96.9%) and full-(*gfp*) (93.0%) comprised nearly the entirety of their respective lysates (Fig. 3). In contrast, M13KO7 was more likely to package itself over target phagemids encoding *cmv-gfp-tet* and *cmv-luc*, regardless of whether the origin was split (miniphagemid), or not (full phagemid). Rescue efficiency of miniphagemids was at least comparable (*cmv-luc*, *cmv-gfp-tet*) or better (*cmv-gfp*) their full phagemid counterparts. Thus, the separation of the hairpins controlling f1 replication was not observed to impede replication at all.

The transgene composition may have some effect on phagemid packaging efficiency. The overall phagemid length appeared to have weak correlation with lysate purity, but it is not likely the key influencing factor. In fact, both *cmv-gfp-tet* phagemids were packaged with a comparable phagemid:helper ratio as the *cmv-luc* phagemids despite their differences in length. All the miniphagemids share the same precursor backbone (pM13ori2) while their full counterparts all share a pBluescript II KS + backbone. The transgene cassettes also share many elements, including the same CMV promoter, polyA sequence, and SV40 enhancer elements. Overall, constructs did not exhibit major differences in base composition; GC content per cassette was almost identical (54%, 46%, 56%, respectively). Indeed, the (*gfp*) and (*gfp-tet*) phagemids were identical, save for the additional 2 kb TetR fragment in the (*gfp-tet*) phagemids, yet this resulted in a stark decrease in phagemid rescue efficiency for both full and mini-(gfp-tet) phagemids. As they all shared the same other non-coding elements, we believe the differences in packaging efficiency must arise from the sequence disparity of the coding regions. Phage production is governed by interactions with three main phage proteins during key steps during the infection lifecycle [7]: (1) pII interaction with the f1 ori, (2) pV coating of the ssDNA molecule, and (3) pI interaction with the packaging signal (Fig. 1). As pV preferentially binds single-stranded DNA, the formation of dsDNA domains may impact its ability to coat the molecule. We postulate that phagemid molecules with higher potential for self-annealing are less efficiently processed into M13 phage particles, independent of phagemid length.

Overall, we have established a robust and straightforward system to produce miniaturized phagemid particles. While the purity of the resultant phage lysate appears to be at least partially dependent on the sequence of the transgene cassette, it is possible to obtain lysates of high titre and almost completely purely phagemid without many downstream processing steps.

### A self-packaging deficient helper phage

**Fig. 4:**
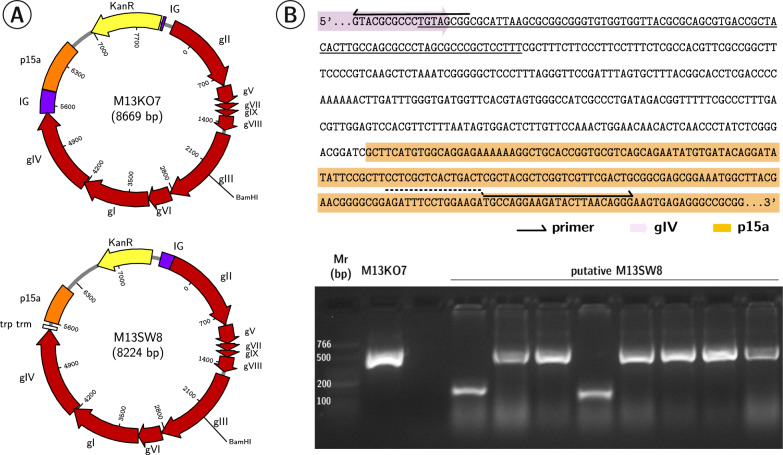
The PS is deleted in M13KO7 to create M13SW8. **A** The intergenic region (IG) containing the packaging signal (PS) between gIV and the p15a *ori* in M13KO7 is deleted to generate M13SW8. **B** The sequence between gIV and the p15a *ori* in M13KO7 is illustrated. Nucleotides identified as part of the PS loop are underlined. Primers for mutagenesis and relevant features are depicted. Below, PCR products of this region are visualized via AGE. From left to right: M13KO7 (386 bp), putative M13SW8 clones (169 bp). Mr: Low Molecular Weight ladder (NEB). AGE: 2.5% agarose in TAE, stained with ethidium bromide

We next constructed a helper phage derivative from M13KO7 (Fig. 4). Levinson et al. [13] postulated that a low phagemid rescue from a helper phage resulted from ssDNA helper intermediates outcompeting phagemid intermediates for pV processing and assembly. Removal of the helper phage PS could decouple helper phage replication from assembly and extrusion. The ∼ 500 bp region containing the filamentous PS between gIV and the p15a *ori* was replaced with a Rho-independent terminator [47] to generate M13SW8 (Fig. 4B). The secondary structure of the PS and other signals in the IR has been implicated in termination of transcription of the preceding gIV coding region [48, 49] so this terminator was placed as a precaution. This filamentous phage would be deficient in its packaging signal but still retain the KanR region in its *ori* region (Fig. 4A). Deletion was verified by PCR amplification of the region between gIV and p15a *ori* region (Fig. 4B). M13SW8 was unable to form visible plaques on susceptible host cells (Table 3) and no detectable levels of ssDNA could be isolated from M13SW8 lysates.

**Table 3:** Phage and colony titres from a helper phage deficient of its packaging signal, *n* = 3

	M13KO7	M13SW8
Phage only		
PA	3.16 × 10^13^	−
CA (Kan)	2.61 × 10^14^	−
Rescue of pBluescript II KS +		
CA (Ap)	4.37 × 10^13^	3.00 × 10^14^
CA (Kan)	4.43 × 10^13^	3.93 × 10^7^
CA (Ap/Kan)	4.53 × 10^13^	3.94 × 10^7^

*PA* Plaque assay (PFU/mL); *CA* Colony assay (CFU/mL), antibiotic indicated in parentheses

### Removal of the packaging signal greatly reduces helper phage self-packaging

Although M13SW8 did not form plaques when plated alone, both helper phages were able to rescue a typical phagemid, pBluescript KS + II. As quantified by colony assay, infective pBluescript phagemid (AmpR) particles were able to confer ampicillin resistance when rescued by either helper (Table 3). Notably, contaminating helper phage conferring KanR was reduced by seven orders of magnitude in M13SW8-rescued lysates compared to M13KO7-rescued lysates without any reduction in corresponding phagemid titres.

Thus, the loss of the PS drastically reduced helper self-packaging. Despite an inability to produce progeny phage particles by itself, PS^−^ M13SW8 infection still occurred when in the presence of the PS^+^ pBluescript phagemid. Single-stranded M13SW8 molecules may simply be assembled into extruding pBluescript particles. Although the PS is required to initiate assembly, phage particle elongation with additional DNA molecules does not appear to have this requirement [50]. In wildtype phage populations, approximately 5% of phage progeny encapsulate multiple genomes [50, 51]. Based on the titres of AmpR and KanR phage from M13SW8-mediated rescue of pBluescript, M13SW8 comprises less than 1% of its lysate. Hence, we postulate that virion production of the PS-deficient helper phage may manifest primarily as multi-length virion particles co-extruded with PS-proficient phagemids.

### Host background impacts phagemid rescue efficiency

At minimum, F-specific filamentous phages have the capacity to infect any Gram-negative bacteria that express cell-surface TolA [52]; for more robust filamentous infection, F^+^ *E. coli* capable of pilus formation are necessary. However, M13 phage replication places an enormous metabolic burden on the infected host. In addition, the presence of a high copy number precursor phagemid further increases the metabolic load due to plasmid replication. As high levels of episomal DNA must be continually maintained, a suitable host should be both proficient in the propagation of large plasmids and M13 phage.

**Fig. 5:**
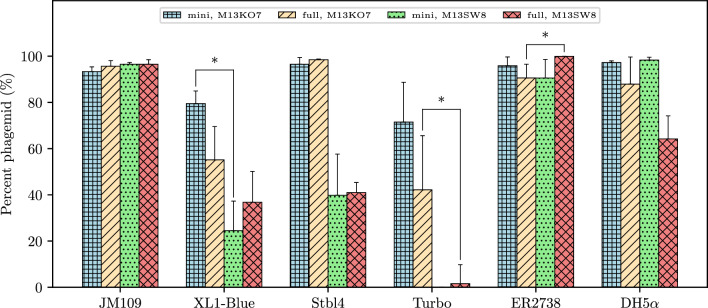
Composition of lysates packaged by PS-deficient helper phage. The proportion of packaged target phagemid (either mini-(*gfp*) or full-(*gfp*)) is shown as a percentage of the total phage population, as produced by either M13KO7 or M13SW8 across 6 different *E. coli strains*. Error bars represent SD, *n* = 3. The ∗ above the bars indicates a difference at significance level *p* < 0.05

**Fig. 6:**
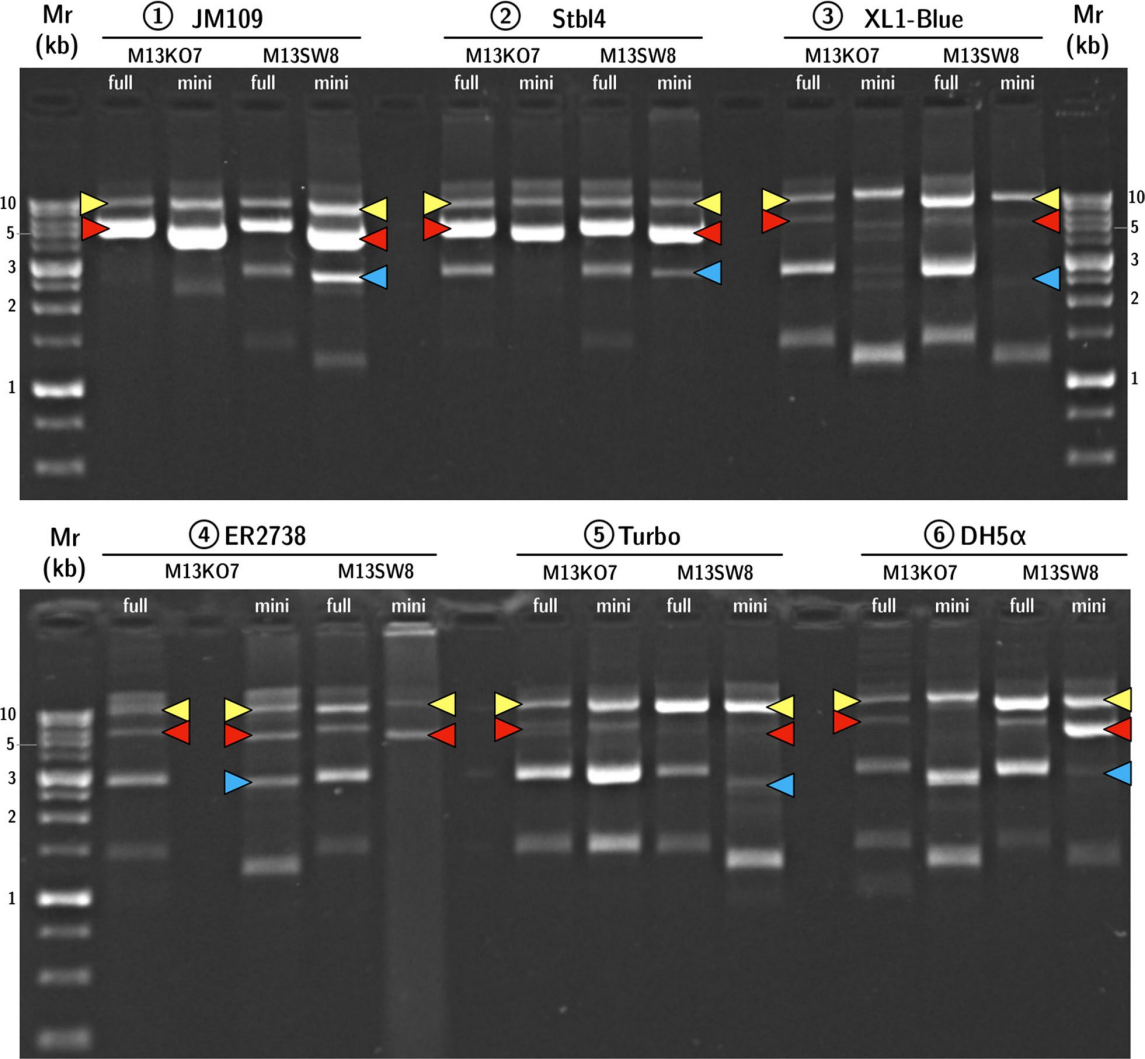
PS-deficient helper phage produces recombinant RF (RFx) across six *E. coli* strains. BamHI-digested RF DNA was extracted from cells carrying either the full or miniphagemid precursor in combination with helper M13KO7 or M13SW8. Top: 1) JM109, 2) Stbl4, and 3) XL1-Blue. Bottom: 4) ER2738, 5) NEB Turbo, and 6) DH5α. Arrows indicate bands of interest: yellow (helper genome), red (plasmid or full phagemid), blue (recombinant RF: RFx). Mr: 1 kb ladder (FroggaBio), AGE: 0.8% agarose in TAE, stained with ethidium bromide

Rescue efficiency was compared between helper phages M13KO7 and M13SW8 in the production of mini-(*gfp*) and full-(*gfp*) across six strains that represent common laboratory strains for plasmid or M13 propagation [53, 54]. The five F^+^ strains were JM109, XL1-Blue, Stbl4, NEB Turbo, and ER2738; an F^−^ control, DH5α, was also compared. Both the helpers and phagemid under investigation have plasmid origins so additional phage transduction or infection may not be necessary for helper phage maintenance. We sought to investigate this using an F^−^ strain as it would minimize infection or re-infection. Each strain was transformed by the mini phagemid precursor pM13ori2.cmvgfp or the full phagemid plasmid (pSW9) and either M13KO7 or M13SW8 to act as the helper. The rescue efficiency was determined from quantification of phage species in each lysate (Fig. 5), while purified RF DNA linearized by BamHI was also visualized via AGE (Fig. 6).

In a head-to-head comparison, M13KO7 was compatible with more host backgrounds than M13SW8 (Fig. 5). Total phagemid yield was also strain-dependent (Table 4). Importantly, both M13KO7 and M13SW8 were able to rescue phagemid in DH5α, despite the lack of an F′ episome indicating that helper plasmids such as these do not require re-infection for high phagemid titres. Indeed, the highest phagemid yield we obtained were from DH5α (Table 4). Interestingly, lysate compositions were consistent across strains (Fig. 5) when M13KO7 was the helper but were highly variable when M13SW8 was used. The increased gene dosage may be resulting in phagemid instability. Overall, M13KO7 and M13SW8 performed very similarly in JM109, ER2738, and DH5α and differed most in Stbl4, XL1-Blue, and NEB Turbo.

**Table 4:** Phagemid titres contrasting helper phage across different host backgrounds, *n* = 3

Strain	Total phage concentration (× 10^8^ gc/μL)			
	M13KO7		M13SW8	
	Mini	Full	Mini	Full
JM109	2.33 ± 0.47	5.24 ± 3.46	5.08 ± 0.21	8.33 ± 2.94
XL1-Blue	2.32 ± 0.36	6.61 ± 2.69	1.29 ± 0.55	2.78 ± 0.67
Stbl4	13.93 ± 5.28	11.77 ± 3.62	0.80 ± 0.40	8.98 ± 3.34
NEB Turbo	1.01 ± 0.04	1.10 ± 0.34	0.78 ± 0.38	0.42 ± 0.10
ER2738	1.38 ± 0.35	6.85 ± 2.59	0.69 ± 0.18	3.85 ± 2.12
DH5ɑ	17.47 ± 16.58	14.72 ± 8.18	1.10 ± 0.58	55.38 ± 47.05

JM109 was the best-performing strain for phagemid purity, with few to no intracellular DNA by-products observed during replication (Fig. 6) and a high phagemid:helper ratio, regardless of helper phage (Fig. 5). ER2738 also proved to be an effective host background for high phagemid production. However, this is a *recA*^+^ strain and examination of the extracted RF shows multiple DNA species in the cell (Fig. 6). Phagemid processing may activate host recombination pathways, producing other DNA species during infection. Accordingly, *recA*^−^ hosts should be preferred over *recA*^+^. Nonetheless, inactivation of *recA* alone is often insufficient to completely eliminate undesired recombination [55]. DNA by-products were observed in other *recA* hosts, like XL1-Blue and Stbl4. Stbl4 is typically recommended for the propagation of unstable inserts or large repetitive or palindromic sequences [56]. M13KO7-mediated rescue of both the mini and full phagemids in Stbl4 were comparable to that of JM109, but M13SW8 performed much more poorly. M13SW8 performance was similarly poor in the other *recA* strain, XL1-Blue, but much better in the *recA* F^−^ strain, DH5α.

In comparison with RF DNA isolated from JM109, purified RF DNA from other strains also showed bands around 1–2 kb (Fig. 6); these bands correlate to ssDNA full-(*gfp*) or mini-(*gfp*) DNA species, i.e. unextruded phagemid DNA. Presence of these species is weak in strain-helper combinations associated with higher phagemid:helper ratios, (ex: JM109), but very strong in the worst-performing strains, XL1-Blue and NEB Turbo. Moreover, the accumulation of such ssDNA intermediates was correlated with M13SW8-mediated rescue across all strains. Such high ssDNA retention within the cell suggests that these molecules were not efficiently assembled into phage progeny, which is reflected in their lower overall yield compared to M13KO7 in some strains (Table 4). On the other hand, phagemid amplification and conversion to RFx appeared to occur readily with the PS-deficient helper across all strains. The rate of assembly and extrusion may be inadequate given the rate of production of ssDNA intermediates. It is possible that increasing the expression of assembly proteins may overcome this issue. Having high intracellular levels of helper phage genome may be interfering with phagemid processing and extrusion, although it is unclear why this occurs in some strains like Stbl4 and XL1-Blue, but not JM109 or DH5α. We offer some possible reasons below for future study.

Plasmid nicking may adversely impact phagemid rescue. In contrast to JM109, the *recA* strain XL1-Blue performed poorly despite its genotypic similarities (Additional file 1: Table S1). While all six strains carry the *endA1* mutation, it has previously been observed that nicking and subsequent plasmid degradation can still occur in *endA* backgrounds [57]. A comparison of multiple *E. coli* strains by Yau et al. [58] found that supercoiled plasmid DNA (5.8 kb) was mostly retained in some *endA* strains like JM107 and DH5α, but was lost in others like NEB Turbo and XL1-Blue. Nicking of supercoiled duplex RF DNA releases DNA gyrase-imposed torsion, leading to a circular relaxed form. Unsupercoiled phage genome is not a template for phage gene expression [60]. This may more strongly impact some phage proteins such as pVIII, which are required in high concentrations and continually depleted during progeny virion production. Furthermore, rolling circle amplification of RFs is normally initiated and terminated by pII binding and nicking specifically within the f1 *ori*. Random nicks on either strand would prevent the synthesis of daughter RFs, reducing the pool of available genome templates for phage gene expression despite high intracellular levels of genome molecules. Host backgrounds that are not truly deficient of endonuclease A would be poor hosts for phagemid production, which may explain the poor performance of XL1-Blue, NEB Turbo and, perhaps, even Stbl4.

Accumulated phage and phagemid genomes may facilitate undesired recombination. Unidentified DNA species were observed in extracted RF of poor strain-helper combinations. The formation of multiple DNA by-products may compete with target phagemids for sequestration and assembly, thereby leading to reduced purity in the phagemid lysate. Notably, a 3 kb DNA species was often detected in extracted RF after helper phage-rescue of full-(*gfp*), but not always after rescue of mini-(*gfp*) (Fig. 6). A phenomenon of “miniphage” formation has been previously observed with infection of filamentous phages at high multiplicity of infection (MOI) [61, 62]. Miniphage by-products primarily consist of only the intergenic region: in both M13KO7 and M13SW8, this region is approximately 3 kb in length i.e., the size of the observed by-product. Since such an intermediate would lack all elements of the phage genome aside from the f1 *ori*, they cannot be a template for phage gene expression. Instead, they would compete against the phagemid for phage resources. Prolonged rates of conversion from intact helper genome to miniphage could eventually suppress gene expression and reduce overall phagemid production. However, it is unclear why this phenomenon should arise only in the case of the full phagemid with an intact origin, and not the split origin miniphagemid precursor.

Earlier, we postulated that M13SW8 presence in the phage lysate may be primarily driven by co-encapsulation with extruding phagemid. This appeared to hold true in high-performing strains such as JM109 and DH5ɑ. However, M13SW8 comprised most of the phage lysate in other strains (specifically, XL1- Blue, NEB Turbo), which appears inconsistent with this hypothesis. It is possible that the accumulation of M13SW8 RF may drive recombination events between phagemid and helper, possibly even reconstituting the PS in the helper at the expense of the phagemid. Such events may be exacerbated with miniphage conversion and random DNA nicking. Furthermore, these strains were also under heavier metabolic burden due to the presence of antibiotic resistance markers [58, 59]. The reduction in overall fitness in these strains creates poor environments for episome stability, which may drive undesired recombination events.

The current study was limited to derivatives of *E. coli* K-12. Other *E. coli* strains may also be attractive as potential hosts, particularly those commonly used in stable plasmid propagation. For example, a phenomenon of unusually high plasmid replication without any decrease in growth rate has been reported in *E. coli* strains TG1, HB101, and MG1655 [58]. These strains may be of particular interest to increase overall phagemid yield. Similarly, the choice of helper phage may also change on the host background. While we focused on M13KO7 as it was previously reported to be highly effective in phagemid rescue [32], we did observe a decrease in rescue efficiency by it and our derivative in some strains. Other helper phages may prove more compatible.

The prevalence of M13-derived mammalian gene delivery systems highlights the immense tuneability of the phage, though many incorporate a transgene directly into the phage genome [1–6]. Notably, another recently reported phagemid system [63] also demonstrated successful production of miniaturized circular single-stranded DNA without bacterial plasmid backbone (cssDNA) by flanking a transgene cassette of interest with modular elements of the f1 *ori*. Unlike the *inho* system, our vector retained all elements of the *ori*, such as the replication enhancer region downstream of the pII nicking site [63, 64], and so, does not require compensatory mutations in the helper phage [65] for high yields. It is clear that minimal phagemid systems like ours and *inho* are heavily dependent on the choice of helper phage or plasmid, in particular, the inhibition of preferential self-packaging by the helper phage, which requires the interruption or deletion of its f1 *ori*.

## Summary and conclusions

The genetic simplicity and flexibility of the filamentous bacteriophage (phage) M13 make it a promising platform for mammalian cell targeting and gene transfer. Overall, the incorporation of two separated domains of the f1 *ori* is a straightforward improvement over a single wildtype origin while still resulting in high titres of miniphagemid gene transfer vectors. As a major stimulator of immunity against plasmid gene delivery vectors is derived from the presence of CpG-rich DNA, the elimination of the phagemid backbone contributes to making a safer, more effective vector. In support of this platform, we constructed a backbone vector, pM13ori2, which contains a polylinker to accommodate cloning of other transgenes, and a novel helper phage with greatly reduced self-packaging. We demonstrated that its use in *E. coli* JM109 can generate very pure phagemid and miniphagemid lysates, without any decrease in yield.

## Supplementary Information

**Additional file 1: Table S1.** Strains used in this study. **Table S2.** qPCR primers to quantify phagemid and helper phage. **Figure S1.** Schematic of pM13ori2. Functional elements of the f1 *ori* are separated by a polylinker. **Figure S2.** Miniphagemid precursor plasmids. Plasmids are derived from pM13ori2. A) pM13ori2.cmvgfp, B) pM13ori2.cmvluc, C) pM13ori2.cmvgfp-tet.** Figure S3.** Full phagemids. Phagemids are derived from pBluescript II KS+. A) pSW9 carries *cmv-gfp*, B) pSW10 carries *cmv-luc*, and C) pSW9-tet is pSW9 plus an additional 2 kb fragment from pBR322.

**Additional file 2.** pM13ori2 sequence.

## Data Availability

All data generated or analysed during this study are included in this published article.
