# Supplementary material for: Construction and characterization of a novel miniaturized filamentous phagemid for targeted mammalian gene transfer
Source: Microb Cell Fact. 2023 Jul 10;22:124. doi: 10.1186/s12934-023-02135-w (PMC10334589; doi:10.1186/s12934-023-02135-w)
Supplement: Supplementary file 1 — Additional file 1: Table S1. Strains used in this study. Table S2. qPCR primers to quantify phagemid and helper phage. Figure S1. Schematic of pM13ori2. Functional elements of the f1 ori are separated by a polylinker. Figure S2. Miniphagemid precursor plasmids. Plasmids are derived from pM13ori2. A) pM13ori2.cmvgfp, B) pM13ori2.cmvluc, C) pM13ori2.cmvgfp-tet. Figure S3. Full phagemids. Phagemids are derived from pBluescript II KS+. A) pSW9 carries cmv-gfp, B) pSW10 carries cmv-luc, and C) pSW9-tet is pSW9 plus an additional 2 kb fragment from pBR322. [file 12934_2023_2135_MOESM1_ESM.docx]

# Additional Information

Table S1 Strains used in this study

| Strain | Genotype | Source |
| --- | --- | --- |
| JM109 | F′ traD36 proAB+ lacI^q^ lacZ‚ ΔM15/Δ(lac-proAB) endA1 glnV44 thi-1 e14^−^ recA1 gyrA96 relA1 hsdR17 | NEB [26] |
| XL1-Blue | F′ traD36 proAB+ lacI^q^ lacZ∆M15 Tn10/ lac endA1 glnV44 thi-1 recA1 gyrA96 relA1 hsdR17 | Agilent Technologies [27] |
| Stbl4 | F′ proAB+ lacI^q^ lacZ∆M15 Tn10/ endA1 glnV44 thi-1 recA1 gyrA96 relA1 ∆(lac-proAB) mcrA ∆(mcrBC-hsdRMS-mrr) λ− gal | Invitrogen |
| NEB Turbo | F′ traD36 proAB^+^ lacI^q^ lacZ∆M15/ ∆(lac-proAB) glnV44 thi-1 galE15 galK16 R(zgb-210::Tn10)TetS endA1 fhuA2 ∆(mcrB-hsdSM)5(r^−^_K_, m^−^_K_) | NEB |
| ER2738 | F′ zzf::Tn10(TetR) proAB^+^ lacI^q^ lacZ∆M15/ ∆(lac-proAB) thi-1 glnV44, ∆(hsdS-mcrB)5, fhuA2 | NEB [28] |
| DH5ɑ | F^−^ φ80 ∆(lacZYA-argF)U169 endA1 thi-1 recA1 gyrA96 relA1 hsdR17(r^−^_K_, m^−^_K_) phoA supE44 | CGSC #12384 [29] |

NEB: New England BioLabs; CGSC: Coli Genetics Stock Center

Table S2 qPCR primers to quantify phagemid and helper phage

| Primer | Amplicon | Sequence (5’ – 3’) | Source |
| --- | --- | --- | --- |
| g5-F | gV | CACCGTTCATCTGTCCTCTTT | [34] |
| g5-R | gV | CGACCTGCTCCATGTTACTTA | [34] |
| gfp-F | *gfp* | CAAGATGAAGAGCACCAAAGG | This study |
| gfp-R | *gfp* | CGAAGTGGTAGAAGCCGTAG | This study |
| luc-F | *luc* | GCGCGGAGGAGTTGTGTT | This study |
| luc-R | *luc* | TCTGATTTTTCTTGCGTCGAGTT | This study |

Figure S1 Schematic of pM13ori2. Functional elements of the f1 *ori* are separated by a polylinker.

Figure S2 Miniphagemid precursor plasmids. Plasmids are derived from pM13ori2. A) pM13ori2.cmvgfp, B) pM13ori2.cmvluc, C) pM13ori2.cmvgfp-tet.

Figure S3 Full phagemids. Phagemids are derived from pBluescript II KS+. A) pSW9 carries *cmv-gfp*, B) pSW10 carries *cmv-luc*, and C) pSW9-tet is pSW9 plus an additional 2 kb fragment from pBR322.
